# Work-related outcomes in randomized, double blind, placebo-controlled trials in osteoarthritis – are they adequately reported in journal publications? A systematic review

**DOI:** 10.1186/s12995-018-0215-8

**Published:** 2018-10-20

**Authors:** Daniel Sowah, Flora Balat, Sebastian Straube

**Affiliations:** Division of Preventive Medicine, Department of Medicine, University of Alberta, 5-30 University Terrace, 8303-112 Street, Edmonton, AB T6G 2T4 Canada

**Keywords:** Systematic review, Search strategies, Treatment trials, Osteoarthritis, Work-related health outcomes

## Abstract

**Background:**

Osteoarthritis (OA) has a high prevalence in Western societies and can affect an individual’s life in a number of domains, including work. In our experience, treatment trials on OA, however, rarely report work-related outcomes. Here we conducted a systematic review to assess the reporting of work-related outcomes in randomized, double blind, placebo-controlled trials in OA. Our systematic review also compared two search strategies for identifying eligible publications, one where work-related terms were included in the database search string (A) and one where this was not the case and work-related outcomes were identified by searches of full text Portable Document Formats (PDFs) (B). Search strategy A would conventionally be used and would only identify publications where work-related terms were mentioned in the title or abstract. Search strategy B presents the innovation of full text PDF searching and would identify publications were work-related terms were reported in the full text, regardless of whether they are mentioned in the title and abstract or not. We hypothesize that search strategy B identifies more relevant publications than search strategy A.

**Methods:**

Electronic database searching was performed in Medline (Pubmed) from database inception to February 23, 2017 to identify peer-reviewed articles of randomized, double blind, placebo-controlled treatment trials in OA of the hand, hip, or knee, available as full-text PDFs. For search strategy A, search terms to identify work-related outcomes were included in the database search string, while search strategy B did not have these terms included in the database search string, but instead involved full text PDF searching. We included English language articles only and only those articles where searchable PDFs were available, to enable a comparison between search strategies A and B. Additionally, included studies also needed to report on pain intensity in relation to the work-related outcomes.

**Results:**

Search strategy A yielded 50 hits combined for hand, hip or knee OA that mentioned some work-related concept in the title or abstract; 12 articles had to be excluded because they were not available as searchable PDFs. Screening of the remaining 38 articles resulted in only two articles that satisfied our inclusion criteria. Search strategy B yielded 986 hits, out of which 201 articles were excluded because searchable full text PDFs were not available. PDF full text searching and further screening resulted in 10 articles that were considered eligible for our review.

**Conclusions:**

Work-related outcomes are rarely reported in journal publication on randomized, double blind, placebo-controlled trials of hand, hip or knee OA. Searching full text PDFs yields more eligible articles than searching titles and abstracts only.

## Background

Osteoarthritis (OA) is a very common condition among the aging population with a high incidence at age 50 years and older [1]. The prevalence estimates of OA vary depending on the specific joint that is affected and the characteristics of the population under study [2, 3]. A study by the World Health Organization (WHO) Scientific Group on Rheumatic Diseases estimated that 10% of the world’s population who are 60 years or older are affected by some form of OA [4]. Based on x-ray imaging, the prevalence of knee OA among an adult cohort aged 56–84 years in Malmo, Sweden, was about 25%, while symptomatic knee OA had a prevalence of 15% [5]. Of the 978 subjects in the Framingham Study Community [6], it was estimated that hip OA was present in 20% of the cohort based on imaging and about 4% had symptomatic OA.

OA is typically characterized by pain, impairment of mobility and stiffness [7]. The pain is related to loss of cartilage, which may lead to joint failure [8, 9]. Factors identified that are associated with OA include inflammation, obesity, weak muscles, cartilage defects and genetic factors [9]. Joints commonly affected by OA include the knee, hips, hand and wrist. Osteoarthritic pain and other accompanying symptoms may lead to a reduction in patients’ quality of life and may also interfere with productivity at the workplace [9]. Indeed, it has been shown that moderate to severe chronic painful conditions may lead to lack of productivity at work [10], work absenteeism as well as substantial financial costs imposed on the healthcare system [10, 11].

For the present review, we refer to outcomes that speak to a person’s ability to work or to productivity at work as ‘work-related outcomes’. Data on such work-related outcomes, which are commonly captured as time lost from work or interference with work ability, are often collected in pain trials – typically as answers to component questions of commonly used questionnaires. However, these work-related outcomes are only infrequently reported in publications on such pain trials [12]. How work-related outcomes in trials on OA specifically have been reported in the scientific literature is not known; this is the subject of the present review.

In this systematic review, we therefore investigated the reporting of work-related outcomes in randomized, double blind, placebo-controlled trials on the treatment of hand, hip or knee OA. Additionally, we had aimed to analyze pain intensity as an outcome in OA trials and to link this pain intensity to work-related outcomes informative of work ability and productivity; however, the data we found did not enable us to conduct dependable analyses on these issues. While there is evidence that work-related outcomes are not frequently reported in journal article abstracts [12], they could conceivably still be reported more often in the full journal articles.

In systematic reviews, electronic database searching involves the use of search terms to identify different concepts and combination of these different concepts. This is followed by screening of the search results based on title and abstract, then full text articles are further assessed, and relevant articles that satisfy the eligibility criteria are included in the review. One major objective of systematic reviews is to capture all (or as large a proportion as possible) of the relevant publications regarding a subject through the database search [13, 14].

The aim of the present publication was to conduct a systematic review employing two distinct search strategies: one where work-related terms were included in the database search string (A) and one where this was not the case and work-related outcomes were identified instead by searches of full text Portable Document Formats (PDFs) (B). Search strategy A would conventionally be used and would only identify publications where work-related terms were mentioned in the title or abstract. Search strategy B presents the innovation of full text PDF searching and would identify publications were work-related terms were reported in the full text, regardless of whether they are mentioned in the title and abstract or not. We hypothesize that search strategy B identifies more relevant publications than search strategy A.

## Methods

### Literature search

**Fig. 1:**
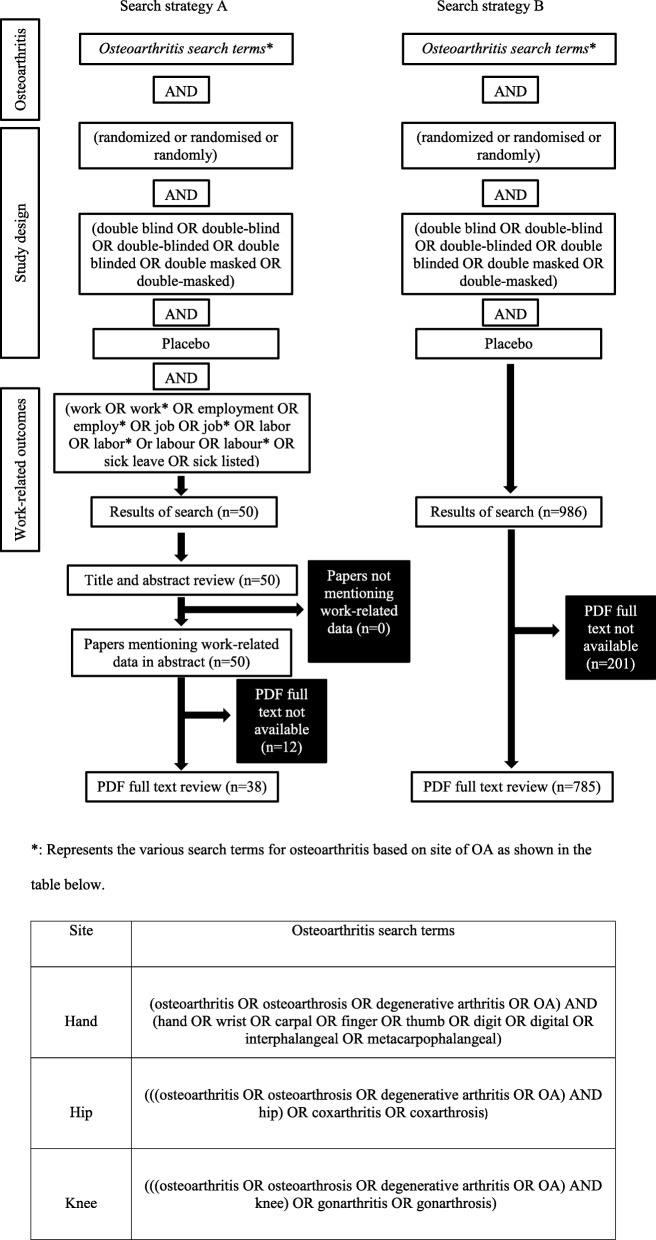
Search strategy and screening of records resulting from two distinct search strategies

Electronic database searches were performed in Medline (Pubmed) from database inception to February 23, 2017. We did not search in a second database, nor did we complement the electronic database search by manual searching. We are aware that this means potentially relevant material was not found, but, to address to aim of the present study, a convenience sample as it was produced by our single database search was deemed sufficient. For search strategy A, search terms to identify work-related outcomes were included in the database search string, while search strategy B did not have these terms included in the database search string, but involved full text PDF searching for work-related terms. The overall approach to searching is shown in Fig. 1.

To be eligible for inclusion in this systematic review, articles must be randomized, double blind, placebo-controlled trials on the treatment of OA of the hand, hip or knee. The publications must be written in English, must report on work-related outcomes, and must be available as searchable PDFs, to enable a comparison between search strategies A and B. Included studies also needed to report on pain intensity in relation to the work-related outcomes.

The search results from search strategy A were screened as abstracts, and publications that did not mention work-related terms were excluded at that stage. The remaining full-text PDFs were further screened to identify articles that satisfied our inclusion criteria. The pathway for search strategy B involved identification of full text PDFs from the search results without restriction to those mentioning work-related terms in the title or abstract, and excluding all abstracts for which the full-text publication could not be found as searchable PDFs. To enable a comparison between search strategies A and B, the articles deemed eligible for inclusion via either search strategy needed to be available to us as searchable PDFs.

When screening the full text journal publication, we searched for the following words, parts of words or phrases in the searchable PDFs to identify those articles that reported work-related outcomes: *work, employ, job, labor, labour, sick leave* or *sick listed*. The screening of the full text articles and selection of included studies was performed by one investigator (FB) and was independently confirmed by another investigator (DS). Discrepancies were resolved by the senior investigator (SS).

### Analysis

Simple proportions of articles reporting outcomes of interest were calculated. No meta-analysis was undertaken as we did not find data suitable for this approach. Had we found such data, we would have used a random effects model or fixed effect model for meta-analysis, as determined by between study heterogeneity (I-squared statistic). We would have calculated odds ratios for dichotomous data and standardized mean differences for continuous data.

## Results

**Fig. 2:**
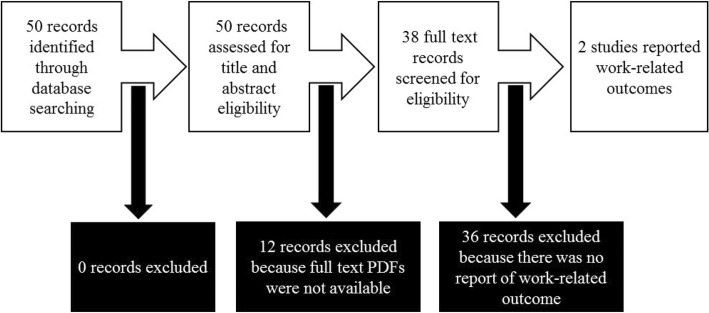
Screening and selection of full text PDF articles of trials reporting work-related outcomes of hand, hip or knee osteoarthritis based on search strategy A. An electronic database search was conducted in Medline (Pubmed) to identify full text articles of randomized, double blind, placebo-controlled trials that reported on work-related outcomes. All articles in which full text PDFs were not available were excluded. The rest were screened and articles that met the requirement for inclusion were selected

Search strategy A yielded 50 hits combined for hand, hip or knee OA, all mentioning some work-related concept in the title or the abstract (Fig. 1). Full text PDFs were not available for 12 papers (24% of the total) and these were therefore excluded. Of the remaining 38 full text PDFs that were screened, only two satisfied our inclusion criteria as shown in Fig. 2.

**Fig. 3:**
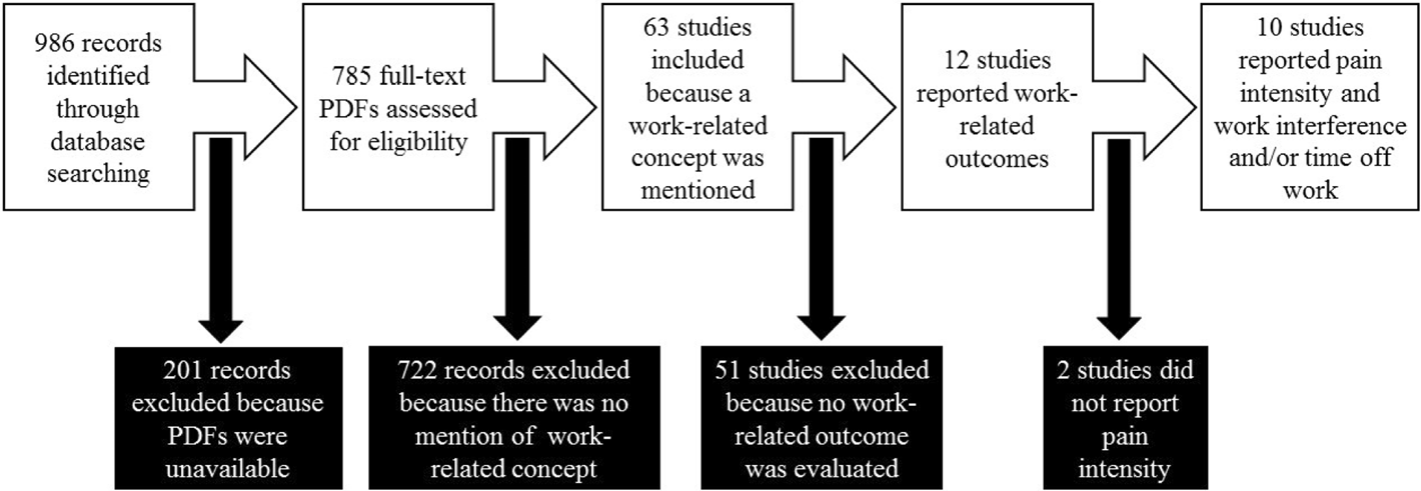
Screening and selection of full text PDF articles of trials reporting work-related outcomes of hand, hip or knee osteoarthritis based on search strategy B. An electronic database search was conducted in Medline (Pubmed) to identify full text articles of randomized, double blind, placebo-controlled trials. All articles in which full text PDFs were not available were excluded. The rest were screened and articles where the full text PDFs mentioned work-related outcome and that met our other requirement were included

Search strategy B generated 986 articles of which there were no duplicates. Of these, 201 records (20% of the total) were excluded because the full text PDFs were not available to us. The remaining 785 full-text articles were screened by electronically searching the PDFs for the work-related terms and verifying the other study characteristics needed for inclusion. This resulted in 722 articles being excluded mainly because the articles did not mention any work-related concept or the articles were not randomized, double blind, placebo-controlled trials. The remaining 63 articles, which mentioned some work-related concept, were further screened to determine which articles actually evaluated and reported work-related concepts associated with OA in the respective study. As shown in Fig. 3, 51 studies were excluded at this stage resulting in 12 studies being retained. Furthermore, two studies [15, 16] did not report on pain intensity in relation to the reported work-related outcomes and were therefore excluded, leading to ten studies that were included in this systematic review. As a tool for assessing the degree of pain associated with the respective OA, six of the included studies [17–22] employed a visual analogue scale (VAS). These studies, however, varied in the work-related outcome measures reported. The work-related outcome measures reported included resumption of work, number of days on sick leave, work performance, pain interference with work and work limitation. Of the four remaining included studies, one study [23] assessed pain intensity using a self-administered, validated questionnaire adopted from the McGill pain questionnaire, the SF-36 Health Survey, and the Medical College of Wisconsin noncancer pain questionnaire. Two of the studies [24, 25] employed the Brief Pain Inventory to assess pain, while one study [26] used a five-point pain scale.

Further comparison between the hits identified with search strategies A and B showed that the ten eligible articles that resulted from search strategy B also included the two eligible articles obtained from search strategy A. Additionally, all the available 38 full text PDFs resulting from search strategy A were also captured by results from search strategy B.

Table 1 lists the 51 excluded articles assessed as full text PDFs and the reasons for their exclusion. Of these 51 excluded articles, five concerned hand OA, 13 hip OA, and 33 knee OA.

**Table 1:** Articles not reporting work-related outcomes in osteoarthritis (excluded after review of full text PDF publications)

Reference	Reason for exclusion
Hand osteoarthritis	
Barthel 2010 [32]	Impact of hand OA mentioned in ‘Discussion’ section, but no work-related outcome was reported
Nieman 2013 [33]	Work disability and inability to work due to knee OA were mentioned in ‘Background’ section, but no work-related outcome was reported
Pope 2004 [34]	Though job status (% working full time, part-time, retired, homemaker) was reported, no work-related outcomes were reported
Richmond 2013 [35]	Number of patients in paid employment reported as part of subjects’ baseline characteristics, but no work-related outcome was reported
Taibi 2009 [36]	Number of patients working mentioned as a part of patients’ baseline characteristics but no work-related outcome measure was reported
Hip osteoarthritis	
Cheras 2010 [37]	‘Work disability’ mentioned in ‘Introduction’ section but no work-related outcome measure was reported for study subjects
Ernst 2003 [38]	Systematic review
Florete 2008 [39]	‘Leading cause of disability; ‘cost’ estimated as ‘lost productive work time’; mentioned as effects of OA in ‘Introduction’ section, but no work-related outcome measure was reported for study subjects
Gana 2006 [40]	‘Leading cause of disability; ‘cost’ estimated as ‘lost productive work time’; mentioned as effects of OA in ‘Introduction’ section, but no work-related outcome measure was reported for study subjects
Jabbari 2011 [41]	Review article
Lambert 2007 [42]	No work-related outcome measure was reported
Maheu 1998 [43]	‘Number or percent of patients’ included in the study and still working was mentioned as a baseline characteristic but no work-related outcome measure was reported
Moskowitz 2003 [44]	Study was a pooled analysis and not a randomized controlled trial
Plosker 2011 [45]	Review article
Pope 2004 [34]	Though job status (% working full time, part-time, retired, homemaker) was mentioned as subjects’ baseline characteristics, no work-related outcome was reported separately
Rozendaal 2005 [46]	Randomized controlled trial study protocol design
Turan 2014 [47]	Loss of labor force mentioned in ‘Introduction’ section only, but no work-related outcome measure was reported
Willich 2010 [48]	Employment status (full/part-time) mentioned as part of patients’ baseline characteristic, but no work-related outcome measure was reported
Knee osteoarthritis	
Alfredo 2011 [49]	Absenteeism due to OA was mentioned in ‘Introduction’ section, but no work-related outcome measure was reported
Auw Yang 2008 [50]	Cost due to loss of productivity at work mentioned in ‘Discussion’ section, but no work-related outcome measure was reported
Babul 2004 [51]	Lost work days due to OA mentioned in ‘Introduction’ section, but no work-related outcome measure was reported
Brown 2013 [52]	Review article
Burch 2007 [53]	Cost due to lost productive work time mentioned in ‘Introduction’ section, but no work-related outcome measure was reported
Cheras 2010 [37]	Costs associated with OA and work disability mentioned in ‘Introduction’ section, but no work-related outcome measure was reported
Cibere 2005 [54]	Work disability and costs associated with knee OA mentioned in ‘Introduction’ section, but no work-related outcome measure was reported
Florete 2008 [39]	‘Leading cause of disability’; ‘cost’ estimated as ‘lost productive work time’; mentioned as effects of OA in ‘Introduction’ section, but no work-related outcome measure was reported
Frakes 2011 [55]	Normal work mentioned as a part of secondary assessment questionnaire due to pain, but no work-related outcome measure was reported separately
Gana 2006 [40]	‘Leading cause of disability’; ‘cost’ estimated as ‘lost productive work time’; mentioned as effects of OA in ‘Introduction’ section, but no work-related outcome measure was reported
Hsieh 2012 [56]	Work status mentioned as a baseline characteristic but no work-related outcome measure was reported
Hsieh 2012 [57]	Work status mentioned as a baseline characteristic but no work-related outcome measure was reported
Hsieh 2012 [58]	Work status mentioned as a baseline characteristic but no work-related outcome measure was reported
Ilan 2004 [59]	Delay in return to work mentioned in ‘Introduction’ section and not reported elsewhere
Jia 2016 [60]	‘Patients maintaining their work activities’ was mentioned in the ‘Discussion’ session, but no outcome measure was reported
Kannus 1992 [61]	Ability to work mentioned as part of assessment of pain due to OA, but no outcome measure was reported
Maheu 1998 [43]	‘Number or percent of patients’ included in the study and still working was mentioned, but no outcome measure was reported
Magrans-Courtney 2011 [62]	Ability to work mentioned as part of assessment of pain due to OA, but no outcome measure was reported
Mata 2015 [63]	‘Reduced participation in work’ mentioned in ‘Introduction’ section, but no outcome measure was reported
Möller 2010 [64]	Work mentioned as part of patients’ assessment tool, but no outcome measure was reported
Mazzuca 2004 [65]	Employment status mentioned as a part of patients’ baseline characteristics, but no outcome measure was reported
Pavelka 1995 [66]	Employment status mentioned as a part of patients’ baseline characteristics, but no outcome measure was reported
Pincus 2001 [67]	Employment status mentioned as a part of patients’ baseline characteristics, but no outcome measure was reported
Pope 2004 [34]	Though job status (% of patients working full time, part-time, retired, homemaker) was mentioned as subjects’ baseline characteristics, no work-related outcome was reported
Sanghi 2013 [68]	Disability leading to work loss mentioned in ‘Introduction’ section, but no outcome measure was reported
Schauss 2012 [69]	Inability to work mentioned in the ‘Methods’ section, but no outcome measure was reported
Segal 2001 [70]	‘Hampers work’ mentioned in ‘Introduction’ section, but no outcome measure was reported
Sihvonen 2013 [71]	Work mentioned as part of patients’ baseline characteristics, but no outcome measure was reported
Singer 2011 [72]	‘Work activities’ mentioned as part of questionnaire to assess quality of life, but no outcome measure was reported
Taverner 2010 [73]	‘Usual work’ mentioned as part of outcome measurement tool, but no outcome measure was reported separately
van der Weegen 2015 [74]	Effect of OA pain on work was mentioned in ‘Introduction’ section; work activities mentioned as part of assessment tool, but no outcome measure was reported separately
Zhao 1999 [75]	Work mentioned as part of quality of life assessment tool, but no outcome measure was reported separately

Although 18 of the 51 excluded studies mentioned some work-related concept in the ‘Introduction’ section of the respective article, they subsequently did not report on any work-related outcome measure of the study participants. The work-related concepts mentioned in the ‘Introduction’ section of the respective article included work disability, inability to work, absenteeism from work, reduced participation in work, decreased work performance, lost productive work time and cost associated with lost work time. None of these work-related concepts were assessed in the participants of the respective studies.

Employment or job status was provided as part of the study participants’ baseline characteristics in 12 studies. The authors of these studies, however, did not report further on any work-related outcomes of the trials. Although some of the excluded studies made mention of some work-related concepts in the ‘Discussion’ and other sections of the articles (21 articles), these were, again, not directly related to the study participants.

Overall, 12 studies reported on the impact of OA and treatments for OA on work-related outcomes related to the study participants (Table 2). One study was on hand OA, two were on hip OA and nine were on knee OA. The particular type of work-related outcomes reported included ‘number of days on sick leave’ (one study) [18], decreased work performance (three studies) [22], limitation of work (four studies) [20–22], pain interference with normal work (five studies) [20–22], and improvement in normal work following intervention (two studies) [22]. In one study [17], resumption of work following therapeutic intervention for hand OA was reported. The ten studies ultimately included in our review (Table 2) reported pain intensity as well as work interference or time off work. The treatments investigated in the ten included studies varied and included naproxen, acetaminophen, celecoxib, rofecoxib, tramadol, duloxetine, soy protein, avocado/soybean unsaponifiables and glycosaminoglycan polysulphate.

**Table 2:** Articles reporting work-related outcomes

Reference	Work-related outcomes reported
Hand osteoarthritis	
Pastinen 1988 [17]	Resumption of work mentioned following treatment of hand OA
Hip osteoarthritis	
Lequesne 2002 [18]	Number of days on sick leave
Smugar 2006 [19]	Decreased work performance
Knee osteoarthritis	
Arjmandi 2004 [23]	Limitation of work due to pain; improvement of work performance and productivity
Chappell 2009 [24]	Pain interference with normal work
Chappell 2011 [25]	Pain interference with normal work
Essex 2012 [20]	Pain interference and work limitation
Golden 2004 [26]	Impact of intervention on work
Hochberg 2011 [21]	Pain interference with normal work
Schein 2008 [22]	Degree of pain interference with work; improvement in work limitation; difficulty in performing work
Schiff 2004 [15]	Improvement in quality of life with regards to work
Wang 2012 [16]	Effect of knee OA on work

All but the final two table entries (Schiff 2004 [15] and Wang 2012 [16]) were included in our systematic review

These ten included articles demonstrated a considerable degree of variability in their reporting of both the work-related outcome measure and pain intensity. This prevented further analysis to determine the correlation between pain intensity and work-related outcome measures, which we had originally planned to do.

In summary, out of the 785 articles that could have reported on work-related outcomes in relation to pain intensity, only 1.3% (10/785) did so.

## Discussion

OA can place a substantial burden on an individual’s life and may lead to reduced productivity at work, interference with work, absenteeism and cost associated with lost work time [27, 28]. Trials on therapies for OA rarely consider the impact of the condition on work-related outcomes. The present systematic review aimed to evaluate the extent to which these work-related outcomes are reported in publications on randomized, double blind, placebo-controlled trials of hand, hip or knee OA. Our systematic review showed that only 1.3% (10/785) of full text articles reported on both, pain intensity (measured mostly by the visual analogue scale) and work interference or time off work.

This finding is consistent with other research, which demonstrated that work-related outcomes in pain trials are infrequently reported based on a database search of abstracts [12]. Chronic pain has been shown to negatively impact work-related outcomes by interfering with productivity and performance at the workplace [29], but this impact is often not captured in the outcomes reported in publications on intervention trials.

In the present review, two different search strategies, strategy A and strategy B, were compared. As described, strategy A followed the conventional search approach, which yielded two articles out of 50 possible identified records, which satisfied our inclusion criteria. By contrast, strategy B, which involved full text PDF searching, generated 986 records out of which ten articles were ultimately eligible. This demonstrates that full text PDF searching may be a useful strategy in circumstances when outcomes of interest are expected to not be reported in abstracts.

The ten included articles demonstrated a high degree of heterogeneity in the reporting of the pain assessment scale employed and the work-related outcome measures reported. Thus, it was not possible to perform any further analysis to determine the association between the pain intensity and work-related outcome measures, which we had originally planned to do. If future studies on OA were to follow similar approaches to assess pain intensity and work-related outcomes, this would enable meta-analysis of data from different studies. Standardization of the outcomes reported would therefore be of benefit.

Although some of the publications collected data on job or employment status of the study participants, the impact on work ability of the pain resulting from OA, and the treatments studied, is most often not reported. We suggest that future trials should put more emphasis on reporting such work-related outcomes in journal publications.

Our findings are limited by the fact that the search was conducted in only one database; potentially relevant articles may therefore have been missed. Stevinson et al. [13] demonstrated that searching only one database was not adequate to capture all possible publications on a given subject. Nonetheless, for our purposes in this systematic review, the results allow us to estimate the extent of under-reporting of work-related outcomes in randomized, double blind, placebo-controlled trials in OA, which is substantial.

Since reduction in pain intensity may lead to an improvement in an individual’s work ability [12], we recommend that treatment trials of OA should routinely report on the work-related outcomes of the study participants. This is in keeping with a recent publication to define an international standard set of outcome measures for patients with hip or knee osteoarthritis, which recommended the reporting of a patient’s work status at baseline and a regular follow-up to evaluate the impact of OA [30]. The recommendations also included various standardized instruments for evaluating pain and impact on overall quality of life. Further, a recent review suggested that core outcomes in OA trials should include time off work (sick leave), employment status, work productivity and work interference [31]. Although most of the included articles in the present review measured either one or more of the above work-related outcomes, the reporting of a particular outcome was inconsistent between the studies. As mentioned above, a standard for work-related outcome measures and reporting that adheres to the same would be goals for the future.

## Conclusions

Work-related outcomes are inadequately reported in journal publication on randomized, double blind, placebo-controlled trials of hand, hip and knee OA. This is an area where future studies can do better.

## Abbreviations

OA: Osteoarthritis
PDF: Portable Document Format
VAS: Visual analogue scale
WHO: World Health Organization
